# Mobility Limitations and Self-Perceived Unmet Dental Needs Among Korean Adults: A Nationwide Multilevel Analysis for Integrated Care

**DOI:** 10.1016/j.identj.2025.109371

**Published:** 2026-01-12

**Authors:** Han-Nah Kim, Nam-Hee Kim

**Affiliations:** a Department of Dental Hygiene, College of Health Science, Kangwon National University, Samcheok, Republic of Korea; b Department of Dental Hygiene, Mirae Campus, Yonsei University, Wonju, Republic of Korea

**Keywords:** Aged, Delivery of health care, Integrated, Dental health services, Health services accessibility, Health service needs, Mobility limitation

## Abstract

**Introduction and aims:**

Self-perceived unmet dental needs reflect access barriers and inequities. In rapidly aging Korea, mobility limitations may exacerbate these barriers, yet evidence disentangling individual, household, and district-level influences is scarce. We examined the association between mobility limitations and self-perceived unmet dental needs and assessed contextual variation.

**Methods:**

We analyzed nationally representative data from the 2024 Korea Community Health Survey (KCHS) for adults aged ≥45 years (n = 167,474). Multilevel logistic regression with district random intercepts estimated adjusted odds ratios (AORs) for self-perceived unmet dental needs, incorporating survey weights and z-standardised district covariates. Absolute differences and prevalence ratios were derived from weighted cross-tabulations, and the intraclass correlation coefficient (ICC) summarised random effects.

**Results:**

Mobility limitations were reported by 20.6%. Unmet needs were more common among those with limitations than without (17.3% vs 11.4%; difference = 5.9 points; prevalence ratio = 1.5). After adjustment, mobility limitations increased the odds of unmet need by about 20% (AOR = 1.2, 95% CI: 1.1-1.3). Need-related factors showed the strongest associations: unmet medical needs (AOR = 5.1), no regular scaling (AOR = 3.0), and chewing difficulty (AOR = 2.5). Between-district variation accounted for 2.3% of total variance (ICC = 0.023). Conventional supply indicators were not significant, while higher oral-health examination rates showed a modest positive association (per 1 SD, AOR = 1.1; *p* = .03).

**Conclusion:**

Mobility limitations constitute an independent barrier beyond socioeconomic and household factors. Persistent unmet need appears driven more by detection–treatment linkage gaps and mobility-related obstacles than by nominal service supply.

**Clinical relevance:**

Mobility-sensitive dental delivery—visiting dentistry, transport support, and assisted referral—may help reduce detection-to-treatment gaps for mobility-limited adults within community-based integrated care.

## Introduction

Oral diseases remain a major global public health challenge, affecting billions of individuals and disproportionately burdening disadvantaged and aging populations.^1^ Korea’s population is aging at one of the fastest rates worldwide, with many older adults concentrated in rural and depopulating municipalities where health and dental infrastructure are diminishing.^2^^,^^3^ For these communities, mobility limitation—a frequent functional impairment in later life—limits not only the ability to visit dental clinics but also the ability to sustain preventive habits and complete treatment, thereby widening inequities in dental care access.^4^

Although socioeconomic disadvantage and poorer self-rated oral health are consistently associated with self-perceived unmet dental needs, these determinants alone do not fully explain observed disparities.^5^^,^^6^ A growing body of evidence indicates that mobility impairments are independently linked to higher odds of self-perceived unmet dental needs, even after accounting for compositional differences.^4^ Earlier studies frequently relied on dentist-to-population ratios or clinic counts as proxies for access, whereas more recent research emphasises “effective access”—the combined influence of supply, demand, travel time, transportation connectivity, and linkage from screening to treatment.^7^, ^8^, ^9^ This distinction is particularly relevant for mobility-limited adults, for whom nominal availability may not equate to usable care.

These challenges intersect with national policy developments. Korea is implementing the Integrated Care Support Act to promote aging in place by integrating medical, dental, and social care at the community level.^10^^,^^11^ Although pilot programs for visiting medical and dental services exist, broader frameworks for home-based dentistry, transport support, and efficient referral systems are still lacking.^12^, ^13^, ^14^ Decision-relevant evidence is therefore needed to quantify how mobility limitations influence self-perceived unmet dental needs and to determine whether conventional supply indicators adequately capture contextual variation in access.

We hypothesised that mobility limitations would remain an independent predictor of self-perceived unmet dental needs after adjustment, that need-related factors would show the strongest associations, and that modest between-district variation would persist beyond conventional supply indicators. The study, therefore, aimed to clarify the association between mobility limitations and self-perceived unmet dental needs across individual, household, and regional contexts.

## Methods

### Study design and reporting

This cross-sectional study used the 2024 Korea Community Health Survey (KCHS), an annual, nationally representative survey administered by the Korea Disease Control and Prevention Agency (KDCA). The KCHS employs a stratified multistage probability design with in-person household interviews.^15^ Reporting followed the STROBE guidelines for observational studies.^16^

### Data sources and setting

Primary sampling units were administrative districts (dong/eup/myeon), and secondary units were households randomly selected within each district.^15^ Individual-level data were drawn from the 2024 KCHS microdata. District-level indicators (si/gun/gu; n = 229) were linked from official administrative statistics.^2^

### Study population

We included adults aged ≥45 years to focus on middle-aged and older adults, as mobility limitations become increasingly prevalent from midlife onward.^4^ This threshold reflects the stage at which age-related functional decline and chronic conditions begin to affect mobility and oral health, when preventive and rehabilitative dental needs become more pronounced. Of 231,728 KCHS respondents, 170,467 were aged ≥45 years. After excluding 1.8% for item nonresponse, the final analytic sample included 167,474 participants residing in 229 districts.

### Exposure (mobility limitation)

Mobility status was determined using the KCHS item, “Do you have difficulty walking?” Responses of “some difficulty” or “bedridden all day” were classified as mobility limitations, whereas “no difficulty” was classified as no limitation. This single-item measure has been widely used in Korean community surveys and aligns with the mobility dimension of the EQ-5D health-related quality-of-life instrument, supporting its construct validity for analyses of functional limitation in oral-health research.^17^

### Outcome (self-perceived unmet dental needs)

Self-perceived unmet dental needs were measured by the KCHS item, “During the past year, did you need dental treatment but not receive it?” (yes/no). This item has been repeatedly used in national epidemiologic studies and shows consistent associations with socioeconomic status, self-rated health, and oral-health behaviors, supporting its construct validity and reliability as an indicator of barriers to care and inequities.^5^^,^^6^

### Covariates

We organised covariates using Andersen’s behavioral model.^18^^,^^19^

• Predisposing factors: sex; age (45-54, 55-64, 65-74, ≥75 years); education (≤elementary, middle, high school, ≥college).
• Enabling factors: economic activity (active vs none) modeled as an individual-level enabling resource; monthly household income treated as a household-level resource facilitating access to care.
• Needs: perceived or expressed needs and health-seeking behaviors, including subjective health (good/poor), subjective oral health (good/poor), chewing difficulty (yes/no), daily toothbrushing (0–1 vs ≥2 times/day), regular dental scaling in the past year (yes/no), unmet medical needs (yes/no), and use of public health facilities (yes/no).^6^^,^^20^^,^^21^
• Household: monthly household income (<1M, 1–<2M, 2–<3M, 3–<4M, ≥4M KRW); marital status (with/without a spouse).
• District level: oral-health examination rate, defined as the proportion of residents in each municipality who received a National Health Insurance covered oral health examination during the previous year. This indicator reflects community-level utilization of preventive dental services and serves as a proxy for regional accessibility to basic oral-health care. The proportion of dental clinics providing oral-health examinations and the number of oral-health examination institutions per 100,000 population represent the supply capacity of preventive oral-health services in the region. In contrast, the number of dental clinics per 100,000 population reflects the overall availability of dental-care resources. For comparability, district-level continuous variables were standardised as z-scores; adjusted odds ratios for these variables are interpreted per 1 SD increase.

### Statistical analysis

We first compared the prevalence of self-perceived unmet dental needs by mobility status using complex-sample chi-square tests, reporting absolute differences (percentage points) and prevalence ratios from weighted cross-tabulations. We then fitted multilevel logistic regression models, with individuals nested within districts (random intercept at the district level), to estimate associations while adjusting for individual-, household-, and district-level covariates.^22^ An unconditional (null) model was estimated to examine between district variation, yielding an intraclass correlation coefficient (ICC) of approximately 0.04, which indicated modest clustering and justified a multilevel framework.^22^ Model fit and parsimony were evaluated using −2 log-likelihood and Akaike information criterion (AIC) values; lower values indicate improved fit across sequential models. Fixed-effect estimates are summarised as AORs with 95% CIs, and district-level heterogeneity is quantified using the ICC. All analyses incorporated the KCHS complex survey design and applied sampling weights as specified in the KCHS documentation.^15^ A 2-sided *p*-value <.05 was considered statistically significant. We also estimated stratified multilevel models by mobility status to facilitate interpretation of effect patterns. To formally test for effect modification, we prespecified 2 interaction terms: (1) mobility limitations × lack of regular dental scaling (binary), and (2) mobility limitations × district-level oral-health examination rate. Because district-level continuous covariates were standardised, the interaction with the examination rate was interpreted per 1 SD increase, consistent with main-effect modeling. Wald tests were used to obtain *p*-values for interaction.^22^ All analyses were conducted using IBM SPSS version 25.0 (IBM Corp.) and R version 4.5.1 (Posit).

### Missing data

Analyses followed a complete-case approach, excluding 1.8% of participants for item nonresponse. Given the small proportion of missing data, a complete-case strategy was considered unlikely to materially bias point estimates; nonetheless, the possibility of bias from differential missingness cannot be excluded.^16^

### Ethics statement

The KCHS is conducted by the KDCA under national ethical oversight, with de-identified microdata provided for public research use. The study was approved by the Institutional Review Board of Yonsei University (IRB No. 1041849-202509-SB-194-01; 2025-09-23).

## Results

### Participants and prevalence

We analyzed 167,474 adults aged ≥45 years from 229 districts; 20.6% reported mobility limitations. The prevalence of self-perceived unmet dental needs was higher among those with mobility limitations than among those without (17.3% vs 11.4%), corresponding to an absolute difference of 5.9 percentage points and a PR of 1.5. These differences were statistically significant in weighted cross-tabulations (Table 1; Supplementary Table S1).

**Table 1:** Distribution of self-perceived unmet dental needs according to mobility status.

Variable	Category	With mobility issue % (95% CI)	No mobility issue % (95% CI)	Absolute difference %p (95% CI)	Prevalence ratio (95% CI)
Overall	-	17.3 (17.2-18.0)	11.4 (11.1-11.5)	5.9 (5.5-6.3)	1.5 (1.5-1.6)
Individual-level					
Predisposing factors					
Sex	Male	17.4 (16.4-18.3)	11.2 (10.9-11.5)	6.2 (5.7-7.2)	1.6 (1.5-1.7)
	Female	18.2 (17.6-18.9)	11.4 (11.1-11.7)	6.8 (6.1-7.5)	1.6 (1.5-1.7)
Age (years)	45–54	26.0 (23.6-28.6)	12.5 (12.0-12.9)	13.5 (11.0-16.0)	2.1 (1.9-2.3)
	55–64	23.7 (22.2-25.2)	12.1 (11.7-12.5)	11.6 (10.0-13.2)	2.0 (1.8-2.1)
	65–74	17.7 (16.7-18.7)	9.8 (9.4-10.2)	7.9 (6.8-9.0)	1.8 (1.7-1.9)
	≥75	15.1 (14.4-15.7)	7.5 (7.1-8.1)	7.6 (6.8-8.4)	2.0 (1.9-2.2)
Education level	≤Elementary	17.4 (16.8-18.1)	11.1 (10.5-11.6)	6.7 (5.6-7.8)	1.6 (1.5-1.7)
	Middle school	18.4 (17.0-19.8)	11.7 (11.1-12.3)	6.7 (5.0-8.4)	1.6 (1.4-1.8)
	High school	19.4 (18.3-20.7)	12.4 (12.0-12.8)	7.0 (5.5-8.5)	1.6 (1.5-1.7)
	≥ College	16.5 (14.9-18.2)	10.2 (9.8-10.6)	6.3 (4.6-8.0)	1.6 (1.4-1.8)
Enabling factors					
Economic activity	Active	19.1 (18.1-20.2)	12.0 (11.7-12.3)	7.1 (5.8-8.4)	1.6 (1.5-1.7)
	None	17.5 (16.9-18.1)	9.9 (9.5-10.2)	7.6 (6.7-8.5)	1.8 (1.7-1.9)
Need factors					
Subjective health	Good	14.5 (14.5-15.3)	10.6 (10.4-10.9)	3.9 (3.4-4.4)	1.4 (1.3-1.4)
	Bad	20.0 (19.3-20.7)	15.8 (15.1-16.5)	4.2 (3.1-5.3)	1.3 (1.2-1.4)
Subjective oral health	Good	10.3 (9.7-11.0)	7.5 (7.3-7.8)	2.8 (2.1-3.5)	1.4 (1.3-1.5)
	Bad	24.1 (23.3-24.8)	21.5 (20.9-22.0)	2.6 (1.6-3.6)	1.1 (1.1-1.2)
Chewing difficulty	No	10.9 (10.3-11.5)	8.7 (8.5-8.9)	2.2 (1.4-3.0)	1.3 (1.0-1.1)
	Yes	27.1 (26.2-28.0)	27.3 (26.5-28.1)	-0.2 (-1.3-0.9)	1.0 (1.0-1.1)
Daily tooth brushing	0–1 times/day	19.6 (18.9-20.4)	13.5 (13.1-13.9)	6.1 (5.0-7.2)	1.5 (1.4-1.6)
	≥2 times /day	16.4 (15.7-17.1)	10.1 (9.8-10.4)	6.3 (5.2-7.4)	1.6 (1.5-1.7)
Regular dental scaling	Yes	9.8 (9.1-10.6)	5.7 (5.5-5.9)	4.1 (3.3-4.9)	1.7 (1.6-1.9)
	No	22.3 (21.6-23.0)	19.2 (18.8-19.7)	3.1 (2.3-3.9)	1.2 (1.1-1.2)
Unmet medical needs	Yes	53.5 (51.2-55.9)	41.3 (39.5-43.2)	12.2 (9.2-15.2)	1.3 (1.2-1.4)
	No	15.0 (14.5-15.5)	10.1 (9.9-10.3)	4.9 (4.2-5.6)	1.5 (1.4-1.5)
Use of public facilities	Yes	17.3 (16.4-18.2)	11.2 (10.7-11.6)	6.1 (4.9-7.3)	1.5 (1.4-1.6)
	No	18.2 (17.6-18.8)	11.3 (11.0-11.6)	6.9 (5.9-7.9)	1.6 (1.5-1.7)
Household level					
Spouse	With	16.0 (15.3-16.7)	10.4 (10.2-10.7)	5.6 (4.9-6.3)	1.5 (1.4-1.6)
	Without	20.3 (19.5-21.1)	14.5 (14.0-15.0)	5.8 (4.9-6.7)	1.4 (1.4-1.5)
Household Income	<1M	21.2 (20.2-22.1)	14.6 (13.7-15.5)	6.6 (5.3-7.9)	1.5 (1.4-1.6)
	1–<2M	18.3 (17.3-19.4)	12.5 (11.9-13.1)	5.8 (4.6-7.0_	1.5 (1.4-1.6)
	2–<3M	16.4 (15.1-17.8)	11.9 (11.3-12.5)	4.5 (2.9-6.1)	1.4 (1.3-1.5)
	3–<4M	15.5 (13.9-17.2)	11.5 (10.9-12.1)	4.0 (2.3-5.7)	1.3 (1.2-1.5)
	≥4M	15.1 (14.0-16.4)	10.5 (10.2-10.8)	4.6 (3.3-5.9)	1.4 (1.3-1.4)

Absolute difference (percentage points) = prevalence with mobility limitations − prevalence without limitations.

• Prevalence ratio (PR) = prevalence with mobility limitations ÷ prevalence without limitations.

• Estimates are from weighted cross-tabulations; p-values are from the complex sample-adjusted chi-square test.

• 95% confidence intervals were estimated using complex survey-adjusted standard errors.

• M = million KRW.

Across sociodemographic and household strata, the pattern was consistent. By education level, absolute differences between adults with and without mobility limitations ranged from 6.3 to 7.6 percentage points. By economic activity, the differences were 7.1 percentage points among the active group and 7.6 percentage points among the none group. Similar gradients were observed across spouse status and household-income categories (Table 1; Supplementary Table S1).

### Multivariable associations (overall model)

**Figure 1:**
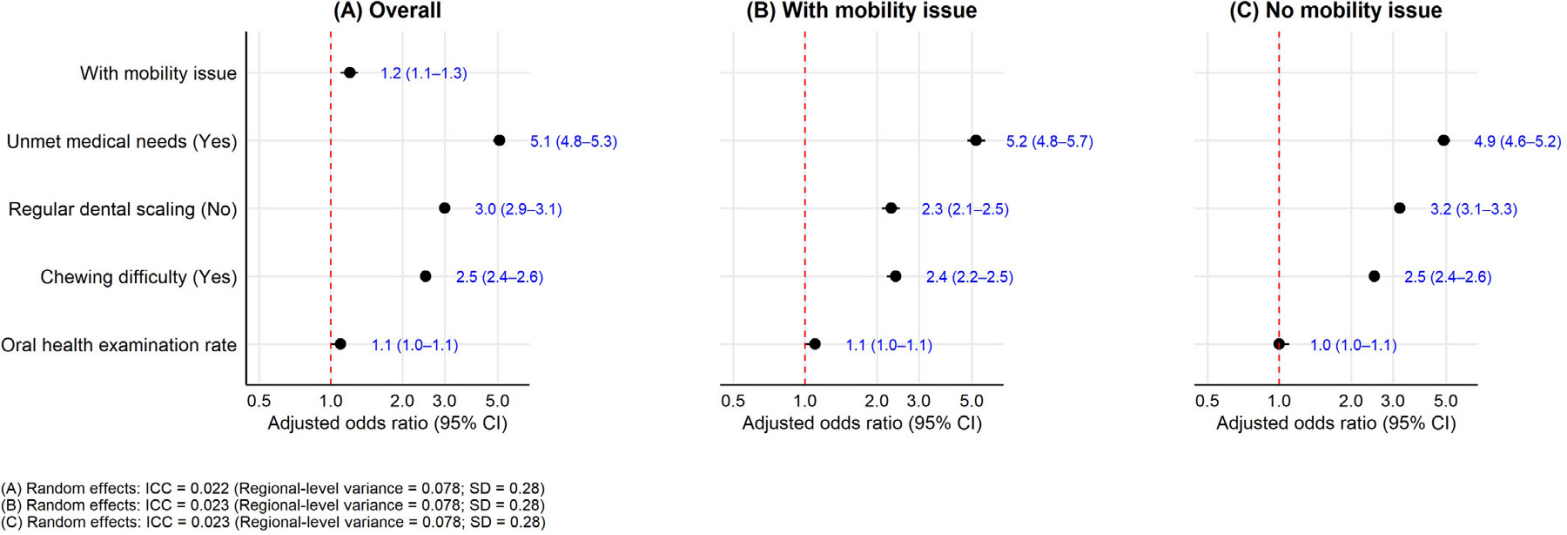
Adjusted odds ratios (AORs) for self-perceived unmet dental needs overall and stratified by mobility status. Dots indicate AORs and horizontal lines 95% CIs. All models are multilevel logistic regressions with district-level random intercepts and survey weights, adjusted for individual, household, and district-level covariates (see Methods). District-level continuous covariates were standardised (z-scores); effects are interpreted per 1 SD increase. Random-effects summaries: ICC ≈ 0.022–0.023. (Panels: A=Overall; B=With mobility issue; C=No mobility issue.).

In multilevel logistic regression models adjusting for individual-, household-, and district-level covariates, mobility limitations increased the odds of reporting self-perceived unmet dental needs by about 20% (AOR = 1.2, 95% CI: 1.1-1.3). Need-related factors markedly increased the odds of self-perceived unmet dental needs: unmet medical needs (AOR = 5.1, 95% CI: 4.8-5.3), absence of regular dental scaling (AOR = 3.0, 95% CI: 2.9-3.1), and chewing difficulty (AOR = 2.5, 95% CI: 2.4-2.6). Social and economic covariates showed modest associations (AOR ≈ 1.1–1.3) (Table 2; Figure 1, Panel A; Supplementary Table S2).

**Table 2:** Adjusted odds ratios for self-perceived unmet dental needs: key individual, household, need, and district-level factors.

Variable	Category	Adjusted OR (95% CI)	p-value
Mobility status	With limitation vs. No limitation	1.2 (1.1–1.3)	<0.001
Unmet medical needs	Yes vs. No	5.1 (4.8–5.3)	<0.001
Regular dental scaling	No vs. Yes	3.0 (2.9–3.1)	<0.001
Chewing difficulty	Yes vs. No	2.5 (2.4–2.6)	<0.001
Regional-level effect	ICC	0.023	—
Oral health examination rate	per 1 SD increase	1.1 (1.0–1.1)	0.03

AOR = adjusted odds ratio; OR = odds ratio; CI = confidence interval; ICC = intraclass correlation coefficient.

• Multilevel logistic regressions with district-level random intercepts and survey design weights applied; covariates include age, sex, education, economic activity, household income, spouse status, need factors (subjective health, subjective oral health, chewing difficulty, tooth brushing, regular scaling, unmet medical needs), and district-level covariates.

• District-level continuous covariates were standardised (z-scores); effects are interpreted per 1 SD increase.

• Reference categories: no mobility limitation; male; age 45–54 years; ≥college education; economically active; with spouse; household income ≥4 million KRW; good subjective health/oral health; no chewing difficulty; brushing ≥2/day; regular scaling in past year; no unmet medical needs; no use of public facilities.

### Random effects and district-level covariates

Random-intercept variance indicated modest but nontrivial between-district heterogeneity (ICC = 0.023). All district-level continuous variables were standardised as z-scores; thus, district-level AORs are interpreted per 1 SD increase. Conventional supply indicators—such as the number of dental clinics or the proportion of providers offering oral-health examinations—did not significantly change the odds of unmet needs after adjustment. The district-level oral-health examination rate showed only a small association overall (AOR ∼1.0-1.1). However, in stratified models, it increased the odds of unmet needs among adults with mobility limitations (AOR = 1.1, 95% CI: 1.0-1.1; *p* = .03) but not among those without limitations (*p* = .22) (Table 2; Supplementary Table S2).

### Models stratified by mobility status

Among participants with mobility limitations, the strongest predictors of self-perceived unmet dental needs were unmet medical needs (AOR = 5.2, 95% CI: 4.8-5.7), chewing difficulty (AOR = 2.4, 95% CI: 2.2-2.5), and absence of regular dental scaling (AOR = 2.3, 95% CI: 2.1-2.5) (Figure 1, Panel B; Supplementary Table S2). Among those without mobility limitations, the corresponding AORs were 4.9 (95% CI: 4.6-5.2), 2.5 (95% CI: 2.4-2.6), and 3.2 (95% CI: 3.1-3.3), respectively (Figure 1, Panel C; Supplementary Table S2). Notably, the association for lack of regular scaling was stronger among adults without mobility limitations than among those with limitations, consistent with the strata-specific AORs (3.2 vs 2.3).

### Interaction tests

Prespecified interaction models corroborated the between-group differences observed in stratified analyses.^22^ The interaction between mobility limitations and absence of regular scaling was significant (AOR = 0.7, 95% CI: 0.6-0.8; *p* < .001), indicating that the association between not receiving regular scaling and self-perceived unmet dental needs was weaker among adults with mobility limitations than among those without—consistent with the strata-specific AORs (2.3 vs 3.2). The cross-level interaction between mobility limitations and the district-level oral-health examination rate (per 1 SD) was also significant (AOR = 1.1, 95% CI: 1.0-1.1; *p* < .001). This aligns with the finding that the examination rate was positively associated with unmet needs in the mobility-limited group (AOR = 1.1, 95% CI: 1.0-1.1; *p* = .03) but not among those without limitations (*p* = .22) (Figure 1; Supplementary Tables S2-S3).

## Discussion

Consistent with our a priori hypotheses, adults with mobility limitations were more likely to report self-perceived unmet dental needs after adjustment. Need-related factors—unmet medical needs, lack of regular scaling, and chewing difficulty—showed the strongest associations. Modest between-district heterogeneity persisted, and patterns were most consistent with a detection-to-treatment linkage gap, especially among adults with mobility limitations.

These findings align with prior work documenting disproportionate dental-care barriers among adults with mobility impairments^4^ and well-established socioeconomic and self-rated oral-health gradients in unmet need in Korea and internationally.^5^^,^^6^,^23^,^24^Our findings align with longitudinal evidence illustrating those persistent social inequalities—rooted in both structural and behavioral factors—ultimately accumulate over time into severe clinical outcomes such as non-functional dentition and tooth loss.^25^

This focus on adults aged ≥45 years is particularly relevant, as this is the period when significant clinical differences in oral health status and the functional impacts of chronic disease begin to emerge and accelerate within the Korean population.^26^ Identifying disparities in self-perceived unmet dental needs during this period may be critical for preventing subsequent tooth loss and functional decline.

The small positive association between district-level oral health examination rates and unmet need—overall and particularly among mobility-limited adults—supports the hypothesis of a detection–treatment linkage gap.^13^^,^^14^ The significant cross-level interaction indicates that these associations were stronger in districts with higher screening intensity, suggesting that increased screening may identify unmet needs that are not effectively translated into completed treatment. This interpretation aligns with calls to integrate oral health within primary and community care and to measure equity-oriented access using metrics beyond nominal counts of services or providers.^12^^,^^27^ In Korea’s predominantly private clinic system, initiatives under the Integrated Care Support Act aim to strengthen coordination between medical and dental sectors for older adults and people with disabilities,^10^^,^^11^ and our findings highlight the importance of extending this coordination from screening to timely treatment.

Null results for traditional supply indicators point to a methodological issue: administrative headcounts do not capture the lived geography of access. Spatial accessibility metrics such as 2-step floating catchment area methods, which integrate supply, demand, and travel impedance, are more policy-relevant and have been linked to preventive care quality/.^7^^,^^8^ Effect-size differences across mobility strata for preventive service use (e.g., larger association between lack of regular scaling and unmet need among adults without mobility limitations) are compatible with a practical ceiling effect, wherein structural barriers diminish the marginal benefit of a single preventive behavior.^28^, ^29^, ^30^

Policy implications follow directly: domiciliary/visiting dentistry with portable equipment, transportation assistance, and navigation systems that link screening to timely treatment should complement any supply expansion; bundling coverage and scheduling to enable same-day or rapid follow-up may mitigate the detection–treatment gap, particularly for mobility-limited adults.^12^^,^^27^ Embedding oral health within community health centers and primary care teams, with task-sharing by dental hygienists, can strengthen continuity and reduce fragmentation.^13^^,^^14^^,^^29^

This study's strengths include the large nationally representative sample, multilevel modeling to partition district-level variance, use of design weights, and triangulation of absolute and relative differences with adjusted associations. Nevertheless, several limitations should be considered. First, the cross-sectional design precludes causal inference and raises the possibility of reverse causality, whereby unmet dental needs could influence perceived health or health-seeking behavior. Second, key variables, including mobility limitation and self-perceived unmet dental needs, were self-reported and thus subject to information bias, including recall error or social desirability bias. Third, selection issues inherent in household surveys may lead to underrepresentation of individuals with severe disabilities or those living in institutional settings, limiting generalizability. Fourth, despite adjustment based on the Andersen model, residual confounding by unmeasured factors, such as detailed clinical oral-health status or psychosocial determinants, cannot be ruled out. Fifth, district-level indicators were based on administrative counts rather than network travel time or effective spatial accessibility; future research in corporation 2-step floating catchment area metrics could provide a more nuanced assessment.^7^^,^^8^ Finally, the absence of marginal predicted probabilities or simple slopes, and the lack of clinical oral-health assessments, may limit the interpretability of effect sizes and the translation into clinical thresholds.

## Conclusion

In a nationally representative multilevel analysis, mobility limitations emerged as an independent barrier to receiving needed dental care in Korea, even after controlling for socioeconomic and household characteristics. Need-related factors—specifically, unmet medical needs, lack of regular scaling, and chewing difficulty—were the strongest correlates of unmet dental care. Regional variation was modest but meaningful: conventional supply metrics were not explanatory, while higher oral health examination rates were modestly associated with greater unmet need among adults with mobility limitations, consistent with a detection–treatment linkage gap. These findings highlight the need for mobility-sensitive, equity-focused strategies, such as domiciliary and visiting dentistry, transportation and navigation supports, and integrated referral-to-treatment pathways, that align oral health services with community-based integrated care for aging populations.

## Data availability

The data that support the findings are available from the Korea Disease Control and Prevention Agency’s KCHS repository; https://chs.kdca.go.kr/chs/rawDta/rawDtaProvdMain.do. Any derived analysis code is available from the corresponding author upon reasonable request.

## Sex and gender considerations

Sex/gender (male/female as defined in KCHS) was included as a covariate; sex/gender-stratified analyses were not a primary objective and are noted as a limitation for generalizability.

## Declaration of generative AI and AI-assisted technologies in the writing process

During the preparation of this work, the authors used ChatGPT **(OpenAI)** for language polishing and consistency checks**.** After using this tool, the authors reviewed and edited the content as needed and take full responsibility for the content of the publication.

## Author contributions

NHK conceptualised and designed the study, developed the methodology, validated the analyses, supervised the research, managed project administration, and contributed to writing – review and editing. HNK contributed to conceptualization and methodology, performed the formal analysis, curated the data, created the visualizations, and drafted the original manuscript. Both NHK and HNK interpreted the results, critically revised the manuscript, verified the underlying data, approved the final version, and agree to be accountable for all aspects of the work.

## Conflict of interest

None disclosed.

## Appendix Supplementary materials

Image, application 1

## Footnotes

Supplementary material associated with this article can be found in the online version at doi:10.1016/j.identj.2025.109371.
