# Supplementary material for: Mobility Limitations and Self-Perceived Unmet Dental Needs Among Korean Adults: A Nationwide Multilevel Analysis for Integrated Care
Source: Int Dent J. 2026 Jan 12;76(2):109371. doi: 10.1016/j.identj.2025.109371 (PMC12828373; doi:10.1016/j.identj.2025.109371)
Supplement: Supplementary file 1 [file mmc1.docx]

| **Table S1. Comparison of self-perceived unmet dental needs according to mobility status: weighted cross‑tabulations by sociodemographic, household, and need factors** | | | | | | | | | | | | | | |
| --- | --- | --- | --- | --- | --- | --- | --- | --- | --- | --- | --- | --- | --- | --- |
|  |  |  | | With mobility issue | | | |  | | No mobility issue | | | |  |
|  |  |  | | Self-perceived unmet dental needs | | | |  | | Self-perceived unmet dental needs | | | |  |
|  |  | Total | | No | | Yes | | p-value | | No | | Yes | | p-value |
|  |  | n | weighted  % | n | weighted  % | n | weighted  % |  | | n | weighted  % | n | weighted  % |  |
| Overall |  | 167,474 | (100.0) | 28,561 | (82.7) | 5,978 | (17.3) |  | 117,727 | | (88.6) | 15,208 | (11.4) |  |
| Individual-level |  |  |  |  |  |  |  |  |  | |  |  |  |  |
| Predisposing factors |  |  |  |  |  |  |  |  |  | |  |  |  |  |
| Sex | Male | 75,076 | (48.0) | 9,100 | (82.6) | 1,700 | (17.4) | 0.124 | 57,030 | | (88.8) | 7,246 | (11.2) | 0.446 |
|  | Female | 92,398 | (52.0) | 19,461 | (81.8) | 4,278 | (18.2) |  | 60,697 | | (88.6) | 7,962 | (11.4) |  |
| Age(years) | 45-54 | 36,445 | (30.9) | 1,054 | (74.0) | 366 | (26.0) | <0.001 | 30,504 | | (87.5) | 4,521 | (12.5) | <0.001 |
|  | 55-64 | 48,658 | (30.9) | 3,271 | (76.3) | 971 | (23.7) |  | 38,763 | | (87.9) | 5,653 | (12.1) |  |
|  | 65-74 | 44,905 | (22.3) | 7,627 | (82.3) | 1,660 | (17.7) |  | 32,045 | | (90.2) | 3,573 | (9.8) |  |
|  | ≥75yr | 37,466 | (15.8) | 16,609 | (84.9) | 2,981 | (15.1) |  | 16,415 | | (92.5) | 1,461 | (7.5) |  |
| Education level | ≤Elementary school | 44,335 | (16.4) | 17,409 | (82.6) | 3,620 | (17.4) | <0.01 | 20,672 | | (88.9) | 2,634 | (11.1) | <0.001 |
|  | Middle school | 24,577 | (12.2) | 4,469 | (81.6) | 903 | (18.4) |  | 16,980 | | (88.3) | 2,225 | (11.7) |  |
|  | High school | 54,557 | (35.5) | 4,571 | (80.6) | 1,036 | (19.4) |  | 42,936 | | (87.6) | 6,014 | (12.4) |  |
|  | ≥College | 44,005 | (35.9) | 2,112 | (83.5) | 419 | (16.5) |  | 37,139 | | (89.8) | 4,335 | (10.2) |  |
| Enabling factors |  |  |  |  |  |  |  |  |  | |  |  |  |  |
| Economic activity | Active | 101,681 | (60.9) | 9,742 | (80.9) | 1,991 | (19.1) | <0.01 | 78,970 | | (88.0) | 10,978 | (12.0) | <0.001 |
|  | None | 65,793 | (39.1) | 18,819 | (82.5) | 3,987 | (17.5) |  | 38,757 | | (90.1) | 4,230 | (9.9) |  |
| Need factors |  |  |  |  |  |  |  |  |  | |  |  |  |  |
| Subjective health | Good | 126,427 | (80.1) | 10,834 | (85.5) | 1,759 | (14.5) | <0.001 | 101,614 | | (89.4) | 12,220 | (10.6) | <0.001 |
|  | Bad | 41,047 | (19.9) | 17,727 | (80.0) | 4,219 | (20.0) |  | 16,113 | | (84.2) | 2,988 | (15.8) |  |
| Subjective oral health | Good | 108,443 | (68.9) | 13,157 | (89.7) | 1,401 | (10.3) | <0.001 | 86,773 | | (92.5) | 7,112 | (7.5) | <0.001 |
|  | Bad | 59,031 | (31.1) | 15,404 | (75.9) | 4,577 | (24.1) |  | 30,954 | | (78.5) | 8,096 | (21.5) |  |
| Chewing difficulty | No | 130,191 | (81.5) | 16,850 | (89.1) | 1,859 | (10.9) | <0.001 | 101,905 | | (91.3) | 9,577 | (8.7) | <0.001 |
|  | Yes | 37,283 | (18.5) | 11,711 | (72.9) | 4,119 | (27.1) |  | 15,822 | | (72.7) | 5,631 | (27.3) |  |
| Daily tooth brushing | 0-1 times/day | 67,374 | (36.7) | 14,262 | (80.4) | 3,167 | (19.6) | <0.001 | 43,201 | | (86.5) | 6,744 | (13.5) | <0.001 |
|  | 2 times/day | 100,100 | (63.3) | 14,299 | (83.6) | 2,811 | (16.4) |  | 74,526 | | (89.9) | 8,464 | (10.1) |  |
| Regular dental scaling | Yes | 81,137 | (55.2) | 8,832 | (90.2) | 981 | (9.8) | <0.001 | 66,959 | | (94.3) | 4,365 | (5.7) | <0.001 |
|  | No | 86,337 | (44.8) | 19,729 | (77.7) | 4,997 | (22.3) |  | 50,768 | | (80.8) | 10,843 | (19.2) |  |
| Unmet medical needs | Yes | 7,490 | (4.4) | 1,254 | (46.5) | 1,381 | (53.5) | <0.001 | 2,825 | | (58.7) | 2,030 | (41.3) | <0.001 |
|  | No | 159,984 | (95.6) | 27,307 | (85.0) | 4,597 | (15.0) |  | 114,902 | | (89.9) | 13,178 | (10.1) |  |
| Use of public health facilities | Yes | 53,181 | (19.8) | 12,373 | (82.7) | 2,474 | (17.3) | 0.104 | 33,939 | | (88.8) | 4,395 | (11.2) | 0.612 |
|  | No | 114,293 | (80.2) | 16,188 | (81.8) | 3,504 | (18.2) |  | 83,788 | | (88.7) | 10,813 | (11.3) |  |
| Household level |  |  |  |  |  |  |  |  |  | |  |  |  |  |
| Spouses | With | 121,012 | (75.1) | 15,178 | (84.0) | 2,815 | (16.0) | <0.01 | 92,090 | | (89.6) | 10,929 | (10.4) | <0.001 |
|  | Without | 46,462 | (24.9) | 13,383 | (79.7) | 3163 | (20.3) |  | 25,637 | | (85.5) | 4,279 | (14.5) |  |
| Household income | <1M | 24,954 | (9.2) | 10,466 | (78.8) | 2,489 | (21.2) | <0.001 | 10,355 | | (85.4) | 1,644 | (14.6) | <0.001 |
|  | 1-<2 M | 29,378 | (13.4) | 7,636 | (81.7) | 1,596 | (18.3) |  | 17,747 | | (87.5) | 2,399 | (12.5) |  |
|  | 2-<3 M | 25,436 | (13.4) | 3,884 | (83.6) | 739 | (16.4) |  | 18,399 | | (88.1) | 2,414 | (11.9) |  |
|  | 3-<4 M | 21,132 | (13.0) | 2,314 | (84.5) | 416 | (15.5) |  | 16,291 | | (88.5) | 2,111 | (11.5) |  |
|  | ≥4 M | 66,574 | (51.0) | 4,261 | (84.9) | 738 | (15.1) |  | 54,935 | | (89.5) | 6,640 | (10.5) |  |
| Si/Gun/Gu-level |  |  |  | **Mean** |  |  | **SD** |  | **Min** | |  |  | **Max** |  |
| Oral health examination rate |  |  |  | 36 |  |  | 13.5 |  | 0 | |  |  | 68.7 |  |
| Prop. Of dental clinics providing oral health examinations | | |  | 65 |  |  | 19.4 |  | 0.0 | |  |  | 100.0 |  |
| No. of oral health examinations institutions per 100,000 population | | | | 21 |  |  | 10.9 |  | 0.0 | |  |  | 93.1 |  |
| No. of dental clinics per 100,000 population | |  |  | 33 |  |  | 15.6 |  | 10.5 | |  |  | 173.8 |  |

Cells show unweighted n and weighted %.
 • p‑values are from the complex sample-adjusted chi‑square test.
 • M = million KRW; SD = Standard deviation; Prop. = Proportion; No. = Number.
 • Variables: oral health examination rate; proportion of dental clinics providing oral health examinations; number of oral health examination institutions per 100,000; number of dental clinics per 100,000.
 • Values are computed at the district level (not survey‑weighted).
 • This table clarifies the scale of 1 SD used for district‑level covariates in regression tables/figures.

| **Supplementary Table S2.** **Full multilevel logistic regression results for self-perceived unmet dental needs (total sample and by mobility status)** | | | | | | | | | | | | | |  |  |
| --- | --- | --- | --- | --- | --- | --- | --- | --- | --- | --- | --- | --- | --- | --- | --- |
|  | |  | Total | | | | With mobility issue | | | | No mobility issue | | | |  |
|  | | | Crude OR  (95% CI) | p-value | Adjusted OR  (95% CI) | p-value | Crude OR  (95% CI) | p-value | Adjusted OR (95% CI) | p-value | Crude OR  (95% CI) | p-value | Adjusted OR  (95% CI) | p-value |  |
| Mobility status | |  |  |  |  |  |  |  |  |  |  |  |  |  |  |
| With limitation  (ref. No limitations) | |  | 1.6(1.6-1.7) | <0.001 | 1.2(1.1-1.3) | <0.001 |  |  |  |  |  |  |  |  |  |
| Fixed effects | |  |  |  |  |  |  |  |  |  |  |  |  |  |  |
| Overall | |  |  |  |  |  |  |  |  |  |  |  |  |  |  |
| Individual-level | |  |  |  | 1.2(1.1-1.3) | <0.001 |  |  |  |  |  |  |  |  |  |
| Predisposing factors | |  |  |  |  |  |  |  |  |  |  |  |  |  |  |
| Sex | | Male | 1.0(reference) |  | 1.0(reference) |  | 1.0(reference) |  | 1.0(reference) |  | 1.0(reference) |  | 1.0(reference) |  |  |
|  | | Female | 1.1(1.1-1.2) | <0.001 | 1.3 (1.2-1.3) | <0.001 | 1.2(1.1-1.3) | <0.001 | 1.4 (1.3-1.5) | <0.001 | 1.0(1.0-1.1) | 0.064 | 1.3 (1.2-1.3) | <0.001 |  |
| Age(years) | | 45-54 | 1.0(reference) |  | 1.0(reference) |  | 1.0(reference) |  | 1.0(reference) |  | 1.0(reference) |  | 1.0(reference) |  |  |
|  | | 55-64 | 1.0(1.0-1.1) | <0.001 | 0.9 (0.9-1.0) | <0.001 | 0.9(0.7-1.0) | <0.001 | 0.8 (0.7-0.9) | <0.01 | 1.0(0.9-1.0) | 0.45 | 0.9 (0.9-1.0) | <0.001 |  |
|  | | 65-74 | 0.9(0.8-0.9) | <0.001 | 0.6 (0.6-0.7) | <0.001 | 0.6(0.6-0.7) | <0.001 | 0.5 (0.5-0.6) | <0.001 | 0.8(0.7-0.8) | <0.001 | 0.6 (0.6-0.7) | <0.001 |  |
|  | | ≥75 | 0.9(0.8-0.9) | <0.001 | 0.4 (0.3-0.4) | <0.001 | 0.5(0.5-0.6) | <0.001 | 0.3 (0.3-0.4) | <0.001 | 0.6(0.6-0.6) | <0.001 | 0.4 (0.3-0.4) | <0.001 |  |
| Education level | | ≤Elementary school | 1.4(1.3-1.4) | <0.001 | 0.8 (0.8-0.9) | <0.01 | 1.0(0.9-1.2) | <0.001 | 0.9 (0.8-1.0) | 0.506 | 1.1(1.0-1.2) | <0.001 | 0.8 (0.7-0.9) | <0.001 |  |
|  | | Middle school | 1.2(1.2-1.3) | <0.001 | 0.9 (0.9-1.0) | <0.05 | 1.0(0.9-1.2) | <0.001 | 1.0 (0.8-1.1) | 0.611 | 1.1(1.1-1.2) | <0.001 | 0.9 (0.9-1.0) | <0.05 |  |
|  | | High school | 1.2(1.2-1.3) | <0.001 | 1.0 (1.0-1.1) | 0.665 | 1.1(1.0-1.3) | <0.001 | 1.1 (0.9-1.2) | 0.157 | 1.2(1.2-1.3) | <0.001 | 1.0 (1.0-1.0) | 0.899 |  |
|  | | ≥College | 1.0(reference) |  | 1.0(reference) |  | 1.0(reference) |  | 1.0(reference) |  | 1.0(reference) |  | 1.0(reference) |  |  |
| Enabling factors | |  |  |  |  |  |  |  |  |  |  |  |  |  |  |
| Economic activity | | Active | 1.0(reference) |  | 1.0(reference) |  | 1.0(reference) |  | 1.0(reference) |  | 1.0(reference) |  | 1.0(reference) |  |  |
|  |  |  |  |  |  |  |  |  |  |  |  |  |  |  |  |
|  | | None | 1.0(0.9-1.0) | 0.111 | 0.8 (0.8-0.9) | <0.001 | 1.0(1.0-1.0) | <0.001 | 0.9 (0.9-1.0) | <0.05 | 0.8(0.8-0.8) | <0.001 | 0.8 (0.8-0.8) | <0.001 |  |
| Need factors | |  |  |  |  |  |  |  |  |  |  |  |  |  |  |
| Subjective health | | Good | 1.0(reference) |  | 1.0(reference) |  | 1.0(reference) |  | 1.0(reference) |  | 1.0(reference) |  | 1.0(reference) |  |  |
|  | | Bad | 1.7(1.7-1.8) | <0.001 | 1.1 (1.1-1.2) | <0.01 | 1.5(1.4-1.6) | <0.001 | 1.1 (1.0-1.2) | <0.05 | 1.5(1.5-1.6) | <0.001 | 1.1 (1.0-1.1) | <0.05 |  |
| Subjective oral health | | Good | 1.0(reference) |  | 1.0(reference) |  | 1.0(reference) |  | 1.0(reference) |  | 1.0(reference) |  | 1.0(reference) |  |  |
|  | | Bad | 3.2(3.1-3.3) | <0.001 | 2.1 (2.0-2.2) | <0.001 | 2.8(2.6-3.0) | <0.001 | 1.7 (1.6-1.9) | <0.001 | 3.2(3.1-3.3) | <0.001 | 2.2 (2.1-2.3) | <0.001 |  |
| Chewing difficulty | | No | 1.0(reference) |  | 1.0(reference) |  | 1.0(reference) |  | 1.0(reference) |  | 1.0(reference) |  | 1.0(reference) |  |  |
|  | | Yes | 3.7(3.6-3.8) | <0.001 | 2.5 (2.4-2.6) | <0.001 | 3.2(3.0-3.4) | <0.001 | 2.4 (2.2-2.5) | <0.001 | 3.8(3.7-3.9) | <0.001 | 2.5 (2.4-2.6) | <0.001 |  |
| Daily tooth brushing | | 0-1 times/day | 1.4(1.3-1.4) | <0.001 | 1.1 (1.1-1.2) | <0.001 | 1.2(1.2-1.3) | <0.001 | 1.1 (1.0-1.1) | <0.05 | 1.4(1.3-1.4) | <0.001 | 1.2 (1.1-1.2) | <0.001 |  |
|  | | 2 times/day | 1.0(reference) |  | 1.0(reference) |  | 1.0(reference) |  | 1.0(reference) |  | 1.0(reference) |  | 1.0(reference) |  |  |
| Regular dental scaling | | Yes | 1.0(reference) |  | 1.0(reference) |  | 1.0(reference) |  | 1.0(reference) |  | 1.0(reference) |  | 1.0(reference) |  |  |
|  | | No | 3.2(3.1-3.3) | <0.001 | 3.0 (2.9-3.1) | <0.001 | 2.3(2.1-2.5) | <0.001 | 2.3 (2.1-2.5) | <0.001 | 3.3(3.2-3.4) | <0.001 | 3.2 (3.1-3.3) | <0.001 |  |
| Unmet medical needs | | Yes | 6.7(6.4-7.0) | <0.001 | 5.1(4.8-5.3) | <0.001 | 6.5(6.0-7.1) | <0.001 | 5.2(4.8-5.7) | <0.001 | 6.3(5.9-6.7) | <0.001 | 4.9(4.6-5.2) | <0.001 |  |
|  | | No | 1.0(reference) |  | 1.0(reference) |  | 1.0(reference) |  | 1.0(reference) |  | 1.0(reference) |  | 1.0(reference) |  |  |
| Use of public health facilities | | Yes | 1.0(0.9-1.0) | <0.05 | 1.0(reference) |  | 1.0(reference) |  | 1.0(reference) |  | 1.0(reference) |  | 1.0(reference) |  |  |
|  | | No | 1.0(reference) |  | 1.0(1.0-1.0) | 0.534 | 1.1(1.0-1.2) | <0.001 | 1.0(1.0-1.1) | 0.44 | 1.0(0.9-1.0) | 0.856 | 1.0(0.9-1.0) | 0.18 |  |
| Household level | |  |  |  | 1.5(1.5-1.6) |  |  |  |  |  | <0.001 |  |  |  |  |
| Spouses | | With | 1.0(reference) |  | 1.0(reference) |  | 1.0(reference) |  | 1.0(reference) |  | 1.0(reference) |  | 1.0(reference) |  |  |
|  | | Without | 1.5(1.4-1.5) | <0.001 | 1.1 (1.1-1.2) | <0.001 | 1.3(1.2-1.4) | <0.001 | 1.1 (1.0-1.1) | 0.07 | 1.4(1.4-1.5) | <0.001 | 1.2 (1.1-1.2) | <0.001 |  |
| Household income | | <1 M | 1.6(1.5-1.7) | <0.001 | 1.2 (1.1-1.2) | <0.001 | 1.4(1.3-1.5) | <0.001 | 1.2 (1.1-1.4) | <0.001 | 1.3(1.2-1.4) | <0.001 | 1.1 (1.0-1.2) | <0.01 |  |
|  | | 1-<2 M | 1.3(1.2-1.3) | <0.001 | 1.1 (1.1-1.2) | <0.001 | 1.2(1.1-1.3) | <0.001 | 1.2 (1.1-1.4) | <0.001 | 1.1(1.1-1.2) | <0.001 | 1.1 (1.0-1.1) | <0.05 |  |
|  | | 2-<3 M | 1.1(1.1-1.2) | <0.001 | 1.1 (1.0-1.2) | <0.001 | 1.1(1.0-1.2) | 0.101 | 1.2 (1.0-1.3) | <0.05 | 1.1(1.0-1.1) | <0.001 | 1.1 (1.0-1.1) | 0.102 |  |
|  | | 3-<4 M | 1.1(1.0-1.1) | <0.001 | 1.1 (1.0-1.1) | <0.01 | 1.0(0.9-1.2) | 0.566 | 1.1 (0.9-1.3) | 0.268 | 1.1(1.0-1.1) | <0.001 | 1.1 (1.0-1.1) | <0.05 |  |
|  | | ≥4 M | 1.0(reference) |  | 1.0(reference) |  | 1.0(reference) |  | 1.0(reference) |  | 1.0(reference) |  | 1.0(reference) |  |  |
| Si/Gun/Gu-level | |  |  |  | 1.7(1.6-1.8) |  |  |  |  |  | <0.001 |  |  |  |  |
| Oral health examination rate | |  | 1.0(1.0-1.0) | <0.001 | 1.0(1.0-1.1) | 0.10 | 1.1(1.0-1.1) | <0.001 | 1.1(1.0-1.1) | 0.03 | 1.0(1.0-1.0) | <0.001 | 1.0(1.0-1.1) | 0.22 |  |
| Prop. Of dental clinics providing oral health examinations | | | 1.0(1.0-1.0) | <0.001 | 1.0(0.9-1.1) | 0.84 | 1.0(1.0-1.0) | 0.47 | 1.0(0.8-1.1) | 0.45 | 1.0(1.0-1.0) | 0.0584 | 1.0(0.9-1.2) | 0.61 |  |
| No. of oral health examinations institutions per 100,000 population | | | 0.9(0.9-1.0) | <0.001 | 1.0(0.9-1.1) | 0.58 | 1.0(1.0-1.0) | 0.19 | 1.2(0.9-1.5) | 0.16 | 0.9(0.9-1.0) | <0.001 | 1.0(0.8-1.2) | 0.83 |  |
| No. of dental clinics per 100,000 population | | | 1.0(0.9-1.0) | <0.001 | 1.0(0.8-1.1) | 0.82 | 1.0(1.0-1.0) | 0.36 | 0.9(0.8-1.1) | 0.35 | 1.0(0.9-1.0) | <0.001 | 1.0(0.9-1.2) | 1.00 |  |
| Random effects | |  |  |  |  |  |  |  |  |  |  |  |  |  |  |
| Variance (Intercept: sigungu) | |  |  |  | 0.078 |  |  |  |  | 0.078 |  |  |  | 0.078 |  |
| SD(Intercept: sigungu) | |  |  |  | 0.28 |  |  |  |  | 0.28 |  |  |  | 0.28 |  |
| ICC | |  |  |  | 0.023 |  |  |  |  | 0.022 |  |  |  | 0.023 |  |
|  | | | | |  |  |  |  |  |  |  |  |  |  |  |

OR = odds ratio; AOR = adjusted odds ratio; CI = confidence interval; ICC = intraclass correlation coefficient; M = million KRW.
 • Models: multilevel logistic regressions with district‑level random intercepts and survey design weights applied; random‑effects estimates for district intercepts (variance, SD, ICC) are shown.
 • District‑level continuous covariates were standardized (z‑scores); effects for these covariates are reported per 1 SD increase.
 • Reference categories as described for Table 2.

**Supplementary Table 3.** Interaction effects of mobility status with regular dental scaling and oral health examination rate on self-perceived unmet dental needs.

| Interaction term | Adjusted OR (95% CI) | p-value |
| --- | --- | --- |
| With mobility issue ✕ Regular dental scaling (No) | 0.7 (0.6–0.8) | <0.001 |
| With mobility issue ✕ Oral health examination rate | 1.1 (1.0–1.1) | <0.001 |

District-level continuous covariates were standardized (z-scores); the interaction with the examination rate is interpreted per 1 SD increase.
